# Topical Antiseptic Formulations for Skin and Soft Tissue Infections

**DOI:** 10.3390/pharmaceutics13040558

**Published:** 2021-04-15

**Authors:** Thi Phuong Nga Hoang, Muhammad Usman Ghori, Barbara R. Conway

**Affiliations:** 1 Department of Pharmacy, School of Applied Sciences, University of Huddersfield, Huddersfield HD1 3DH, UK; thiphuong.hoang@hud.ac.uk (T.P.N.H.); m.ghori@hud.ac.uk (M.U.G.); 2 Institute of Skin Integrity and Infections Prevention, University of Huddersfield, Huddersfield HD1 3DH, UK

**Keywords:** skin, soft tissue infections, antiseptic, nanocarriers, formulations, nanoparticles

## Abstract

Skin and soft tissue infections (SSTIs) are usually acute conditions of inflammatory microbial occupation of the skin layers and underlying soft tissues. SSTIs are one of the most frequent types of infection, typically requiring medical intervention and contribute to morbidity and mortality in both primary care and hospitalised patients. Due to the dramatic rise of antibiotic resistance, antiseptic agents can be potential alternatives for the prevention and treatment of SSTIs. Notably, they are commonly recommended in many global practical guidelines for use in per- and post- operative procedures. A range of antiseptics, including chlorhexidine, triclosan, alcohol, and povidone-iodine, are used and are mainly formulated as traditional, simple dosage forms such as solutions and semi-solids. However, in recent years, there have been studies reporting the potential for nanotechnology in the delivery of antiseptics. In this review, we have collated the scientific literature that focuses on topical antiseptic formulations for prevention and treatment of SSTIs, and have divided findings into traditional and advanced formulations. We conclude that although nanotechnological formulations have demonstrated potential advantages for delivering drugs; nevertheless, there is still scope for traditional formulations and further development of optimised topical formulations to address the rise of antimicrobial resistance.

## 1. Skin and Soft Tissue Infections

Skin and soft tissue infections (SSTIs) refer to acute conditions of inflammatory microbial occupation of the skin layers and underlying soft tissues [1,2]. The consequences have implications on healthcare, not only in low and middle income countries, but also globally [3]. SSTIs, is considered as one of the most frequent types of infection, typically require medical intervention and contribute to morbidity and mortality in both primary care and hospitalised patients [2]. It is estimated that 7–10% of hospital administrations in North America in 2005 were as a consequence of skin and soft tissue infections [4]. In the United States there was an increase of 65% in patients admitted with SSTIs in different hospital departments, from 32.1 visits per 1000 population in 1997 to 48.1 visits per 1000 population in 2005 [5]. Likewise, Lee and co-workers surveyed SSTIs occurrence in the US from 2000 to 2012 and reported that the total prevalence of SSTIs rose from 2.4 million to 3.3 million (an increase of nearly 40%) during this period [6]. In 2013, almost a third of the US population asked for medical advice related to skin conditions [7]. The incidence of SSTIs has increased, possibly as a result of an ageing population, the escalation of multidrug-resistant strains and the increasing numbers of immunocompromised patients as a consequence of immunosuppressive therapy, cancer, transplant interventions, or HIV⁄AIDS [2,8]. Global Health Metrics reported in 2017 regarding the prevalence, incidence, and years lived with disability (YLDs) covering 354 diseases in 195 countries; accordingly, there were nearly 4.2 billion new cases of skin and subcutaneous diseases worldwide. Around 50% of these were fungal skin diseases (accounting for more than 2.1 billion), whereas the incident cases linked with bacterial and viral pathogens were 0.27 and 0.12 billion, respectively [9].

Pathophysiology of SSTIs is related to an interruption in the balance between the immune barrier of the host and the pathogenicity of microbial population colonizing human skin [2]. Cellulitis, as an example, is caused by pathogens disrupting skin integrity, and is more prevalent in patients with comorbidities [10]. Disruption of the protective cutaneous layers can be caused by chemical and physical impacts such as ulceration, trauma, bites or surgical wounds, thermal injury, or previous inflammation [2,10]. Both the patient and the environment are key factors contributing to the risk of developing an SSTI. Older people or those with long-term conditions such as critical illness, obesity, cardiovascular diseases, and chronic kidney disease failure will be at higher risk of skin breakdown. Patients with spinal cord injury and paralysis that result in the alteration of skin perfusion and temperature control are also considered to be at higher risk. External factors which are likely to impair the skin barrier function can be scratching, pressure, shear and friction, UV exposure, or radiation contact in cancer patients [2,11]. Additionally, biofilm formation, the development of which enables microbes to survive and adapt to unfavourable conditions, has become a severe problem in the healthcare fields, responsible for 65% of nosocomial infections [12]. Biofilm is produced following a cell attaching to a surface, multiplying, maturating, and then creating an extracellular polymeric matrix which resists environmental impacts such as mechanical forces and antibiotics. This structure is detachable, affording opportunities for microorganisms to transmit into new sites and spread infections. Biofilms have been observed in medical devices such as intravenous and urinary catheters, stents, implants, ventilator tubes, or heart valves, contributing to the growing challenge of antimicrobial resistance [13].

In children, bacterial skin infections are more prevalent than fungal, parasitic and viral infections [14]. The major causative pathogens associated with skin and soft tissue infections are Gram-positive microorganisms, typically *Staphylococcus aureus* (including methicillin-resistant *S. aureus*/MRSA strains) and beta-hemolytic streptococci [1]. The most frequent Gram-negative strain isolated was *Klebsiella* sp. [15]. *S. aureus* was responsible for more than 40% of total SSTIs cases in 2003, and was a frequent cause of cellulitis, abscesses and wound infections [2]. The incidence of *S. aureus*-related skin and soft tissue infections increased two-fold from 2001 to 2009 in the US [16]. However, it was reported that the proportion of hospital administrations caused by MRSA-related skin and soft tissue infections (SSTIs) declined by 29% over the next five years [17].

Patients with dermatologic conditions often encounter physiological, psychological, as well as financial issues; not only that, many cutaneous concerns can lead to systemic diseases [18]. Moreover, comorbidity factors, such as diabetes, immuno-compromisation, obesity, liver and kidney failure, and cardiovascular diseases, have repercussions on treatment costs and prolong the length of stay in hospital [19]. Suaya et al. determined that the cost of SSTI hospitalizations due to *S. aureus* in 2009 was $4.50 billion, which was 34% higher than in 2001 [16]. According to the Global Burden of Disease Study, 15 different dermatologic concerns accounted for 1.79% of the total global burden of disease in 2013. This was calculated using disability-adjusted life years (DALYs) index, of which cellulitis, viral skin diseases and fungal skin diseases accounted for 0.04%, 0.16%, and 0.15%, respectively. Skin and subcutaneous conditions, next to iron deficiency anaemia, tuberculosis, and sensory organ diseases were the leading reasons inducing disability in the world [9,18].

The management of SSTIs often depends on the relative severity. Uncomplicated SSTIs, located in superficial layers, typically can be controlled with a topical antimicrobial agent, heat packs or minor incision and wound exudate draining, while more complicated cases with involvement of deeper layers with high-risk factors often require systemic antibiotic therapy and hospital administration [2]. With regards to the emergence of resistant bacteria and antimicrobial stewardship, there is an overall drive to reduce any unnecessary and inappropriate use of antibiotics. Owing to the broad-spectrum of antimicrobial activity alongside with the varying inhibitory mechanisms, topical antiseptics are advocated as a potential alternative to topical antibiotics in the treatment of minor skin infections [20,21,22]. Although the safety and clinical effectiveness of many antiseptic agents have not been widely demonstrated so far [23], they bring potential benefits in the prevention of infections in wounds [20] and are still commonly recommended during pre- and per-operative processes which are documented in many global practical guidelines [24]. Further, a wide range of antiseptics are used mainly as simple dosage forms like solutions and semi-solids, but there have been numerous studies to implement formulation strategies in order to potentially influence therapeutic efficacy in recent years [25]. Hence, the purpose of this review paper is to collate the studies involving formulation of antiseptics for application via the topical route in the prevention and treatment of skin and soft tissue infections.

## 2. Antiseptics

**Figure 1:**
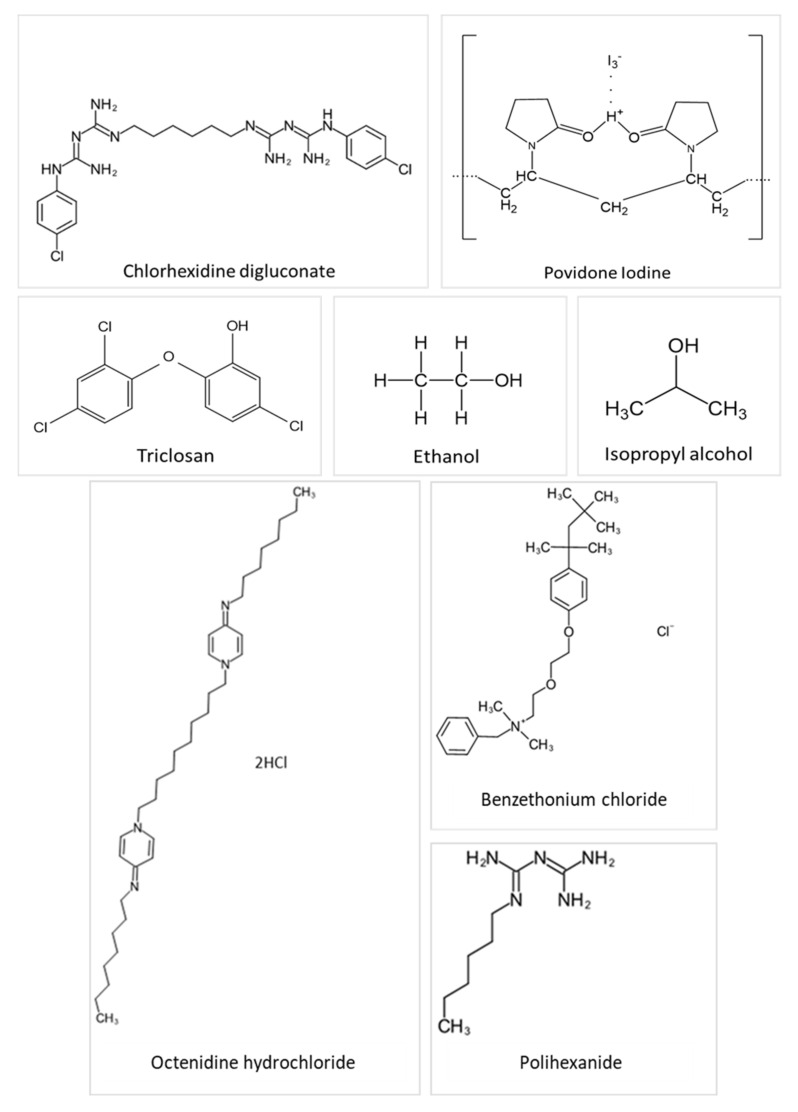
Chemical structures of several antiseptic agents.

Antiseptics are biocidal products that can kill or impact the growth of disease-causing bacteria in, or on, living tissue, e.g., on the skin. Ideal properties for antiseptics include widespread and rapid bioactivity against bacteria, fungi and viruses, no toxicity or damage to the healthy tissue, and insignificant absorption into the systemic circulation following external application [26]. Antiseptic products may contain one or more active ingredients and are presented in various formulations and preparations, for example, antimicrobial hand washes, surgical scrubs, preoperative preparations, tinctures, ointments, creams, mouth-rinses, and toothpaste. They are commonly used as pre-operative skin preparations for prevention of surgical site infections [27], as routine skin hygiene such as hand-washes and hand rub products or for treating skin and wound diseases [26]. For skin and wound infections in deeper skin layers, antibiotics are more normally prescribed; in contrast, topical antiseptics are preferred for infections at the outermost surface. In such cases, the aim is to minimize any microbial colonization in a wound or on the skin surface without causing any deleterious effects on the living tissue or impeding the healing process [26,28]. Chemical structures of commonly used antiseptics are depicted in Figure 1.

### 2.1. Chlorhexidine

Chlorhexidine is a cationic polybiguanide (bisbiguanide) [29]. It primarily used as salt forms because of its insolubility in water. Chlorhexidine gluconate (CHG) and other salts like chlorhexidine diacetate, dihydrochloride, and dihydrobromide are used as surficial disinfectants (disinfection of the skin and hands), in cosmetics (in creams, toothpaste, hair care products, deodorants, and antiperspirants), and pharmaceutical products (e.g., preservative in eye drops, wound dressings, and antiseptic mouth-rinse) [26]. Chlorhexidine is supplied typically in solution from 0.5 to 4% *w*/*v*. Chlorhexidine gluconate 2% *w*/*v* (CHG) in 70% *v*/*v* isopropyl alcohol (IPA) is particularly recommended for pre-operative skin cleansing by several organizations, such as Health Protection Scotland (2013), the Centre for Disease Prevention and Control (2017), National Institute for Health and Clinical Excellence (2013) and the World Health Organization (2017) [30,31]. Chlorhexidine solutions at concentrations of 0.5% *w*/*v* and above, with alcohol, are employed to prepare skin prior to peripheral venous catheter insertion to prevent catheter-related bloodstream infections [32]. Chlorhexidine is a broad-spectrum antibacterial, active against both Gram-positive and Gram-negative bacteria, while exhibiting some activity on yeasts, dermatophytes, and some lipid-enveloped viruses [26]. Furthermore, Macias et al. concluded that CHG in IPA is preferred to 1% *w*/*v* triclosan in 70% IPA when a prolonged antisepsis is required due to its longer-lasting residual effect, [33]. Alcoholic CHG solutions at both 0.5% and 1.0% *w*/*v* concentrations were better than 10% *w*/*v* aqueous povidone-iodine (PVP-I) in minimizing microbial colony formation related to intravascular catheters [34]. Gels containing 2% *w*/*v* CHG also demonstrated a higher fungicidal activity than a comparative nanosilver gel against *C. albicans* [35].

The mechanism of antimicrobial activity of chlorhexidine is that the positively charged molecule binds to the negatively charged lipid bacterial cell surface, thus weakening the cell membrane integrity, followed by leakage of cytoplasm and precipitation of proteins and nucleic acids at lower concentrations and membrane disruption at higher concentrations [26,36]. Due to this non-specific mechanism of action, chlorhexidine use is widespread. However, there are some issues with its use, such as potential toxicity in the eyes, ears and brain, it can become inactivated in the presence of non-ionic surfactants, and it may cause dry skin [26,37]. Recently, the Food and Drug Administration (FDA) released a warning regarding the increasing occurrence of rare but severe allergic reactions to CHG. According to the FDA, healthcare specialists should take into account the patient’s allergy history prior to prescribing CHG-based products [38]. Furthermore, some recent studies have indicated that the increased use of CHG may be responsible for cross-resistance to colistin and daptomycin and the reduced susceptibility (manifested by higher CHG minimum inhibitory concentrations) against several skin pathogens such as *Klebsiella pneumoniae*, multidrug-resistant *Acinetobacter baumannii*, *S. epidermidis, S. aureus,* and vancomycin-resistant enterococci (VRE) [39,40,41,42].

### 2.2. Triclosan

Triclosan (2,4,4′-trichloro-2′-hydroxydiphenyl ether) is a phenoxyphenol compound that has been principally considered as an antibacterial and antifungal agent [26]. Triclosan has a very low aqueous solubility of 0.012 g/L at 20 °C [43]. It is a common ingredient in various antiseptic products, especially in antimicrobial soap, body and hand washes and toothpaste. It is typically used at concentrations of 0.1 to 2% *w*/*v*, with or without other active antimicrobials such as alcohols, to bring about long-lasting activity on the skin. Triclosan is active against Gram-positive bacteria, including *Staphylococcus* species. Moreover, it may also have an effect on Gram-negative bacteria and yeast, with some weaker activity against enveloped viruses, pseudomonads, and fungi [26]. Originally, triclosan was thought to target the cell membrane in a non-specific mechanism, however, recent studies have found a specific bacteriostatic action for triclosan on bacteria through inhibition of the bacterial fatty acid biosynthetic pathway. At the higher concentrations found in antiseptic preparations (2–20 mg/mL), there is a hypothesis that triclosan acts as a biocide with multiple actions on lipid, RNA and protein synthesis, leading to cell lysis [42,44]. The antimicrobial activity of triclosan-containing antiseptics can be influenced by formulation effects, for example, there is a synergistic activity with chelating agents (e.g., EDTA) in destroying the Gram-negative bacterial cell wall thereby improving uptake into cells. Triclosan shows negligible irritation and allergic skin reactions and it can retain persistent on the skin surface [26]. However, because of the lack of the scientific literature regarding the safety and effectiveness of triclosan for human health, in December 2017, the FDA issued a final rule prohibiting the use of triclosan in certain over-the-counter antiseptic preparations [23].

### 2.3. Povidone-Iodine (PVP-I)

Povidone-iodine (PVP-I), which is a complex of elemental iodine loosely bound to the carrier polyvinylpyrrolidone, is used as a broad-spectrum antimicrobial agent against bacteria, viruses, fungi, and protozoa at relatively low concentrations [26,45]. Typically, PVP-I is widely used as a topical antiseptic and disinfectant for skin and wound infections, mostly in solution, dry powder and lotion formulations. Application as an iodophor improves both solubility and stability while releasing the active iodine gradually from the polymer network over time. Therefore, its residual antimicrobial activity is maintained stably while side effects associated with iodine such as irritation and brown staining on the skin and mucous membranes are reduced. Its precise mechanism of action is still unknown, but it is believed that the active iodine species acts as an oxidizing agent which reacts with cell walls, membranes, and cytoplasm by exchanging and inactivating functional groups of amino acids (e.g., lysine, histidine, cysteine, and arginine). The consequence is the loss of cell structure and function [26].

### 2.4. Alcohol

Alcohols offer rapid and broad-ranging activity against bacteria, fungi, and viruses although less is known about their activity against protozoa and bacterial spores, but they are sporistatic. Isopropanol (isopropyl alcohol), ethanol and *n-*propanol are the most popular alcohols used as antiseptics and disinfectants. Their exact mechanism of action is not clear but they are able to cause denaturation and precipitation of proteins thus destroying cell membranes and leading to cell lysis. Concentrations ranging from 60% to 80% *v*/*v* are recommended for maximum antimicrobial activity because, in more concentrated solutions, alcohol quickly coagulates protein-based molecules present externally on the cell wall and interferes with penetration into the cell, therefore limiting further effects on protein-based inner cell compositions. Other potential attributes are relative stability, and low toxicity, odour and cost. Alcohols are also used as preservatives and common solvents for other biocides such as chlorhexidine [26].

### 2.5. Essential Oils

Essential oils are secondary metabolic products found in various parts of plants (such as flowers, seeds, leaves, peels, buds, barks, wood, or roots), and can be extracted by hydro-distillation and steam distillation, mechanical processes, or by “dry” distillation from some woods [26,46]. They are complex mixtures containing hundreds of compounds and their exact chemical composition depends on extraction processes and specific conditions. For example, dry vapour steam distillation is used when there is a requirement to minimize ester hydrolysis (e.g., linalyl acetate), or cohobating is proposed to improve the quantity of particular compounds such as sulfur compounds [46]. Essential oils and their components have been used in a wide range of products, from fragrances, toothpastes, cosmetics, to aromatherapy and phytomedicine, with tea tree oil and eugenol, being combined in many commercial antiseptic preparations, such as Ord River Tea Tree Antiseptic Cream^®^, Australian Tea Tree Antiseptic Cream^®^ or Manuka Doctor ApiRevive Manuka and Tea Tree Antiseptic Gel^®^ [26]. In dermatology, essential oils are primarily used for treating skin infections (62% of total cases), followed by skin inflammation and general skin maintenance at 20% and 18%, respectively [47]. Relative bioactivity varies between the different oils. In particular, tea tree oil demonstrates bactericidal activity (at 0.25 to 0.5% *v*/*v*), fungicidal activity (at 0.06–1% *v*/*v*), fungistatic activity (within 0.03–0.5% *v*/*v*) as well as activity against yeasts and dermatophytes (including *Candida* and *Trichophyton*). Tea tree oil, amongst others, presents persistent and long-lasting activity on the skin after application. Despite most essential oils presenting antimicrobial effectiveness at low concentrations, they have been reported to generate irritancy and allergenicity following application to skin and mucous membranes [26,47]. Almost 1.8% of patients tested with 5% and 10% tea tree oil patches experienced allergic contact dermatitis [48].

### 2.6. Silver Compounds

The active element is the silver ion (Ag^2+^) in silver nitrate (AgNO_3_) and silver sulfadiazine (AgSD). Generally, topical silver antiseptics are applied for prevention of skin and wound infections mostly caused by *S. aureus* and *Pseudomonas* in cream or solution forms and used in eye drop preparations for bacterial infections in neonates [26]. There are a number of studies indicating the valuable role of silver in wound care [49]. Additionally, silver compounds are also commonly used to cover surfaces prone to bacterial colonization such as catheters or dental instruments. Many commercial silver-based products are now available in many forms such as Atrauman Ag^®^ Wound Dressing, Urgotul^®^ SSD Antibacterial Contact Layer, Flamazine^®^ Antibacterial Cream, Colloidal Silver Spray^®^, Silver Solution^®^ Antimicrobial Wound Gel, MSM+Silver^®^ Water Drops, or Natural Sense Colloidal Silver ^®^ Eye Drops.

Silver compounds exhibit bacteriostatic and bactericidal activity at fairly low concentrations, especially on Gram-positive bacteria. Regarding the mechanism of action, active silver ions bind to sulfhydryl, amino, and carboxyl groups of amino acids on microorganism surfaces, thus denaturing proteins, and disrupting the cell wall and membrane functions. Silver also specifically inhibits cell wall metabolism and electron transport as well as the respiration chain [26,50]. Following application of topical antiseptic, respiratory sprays, implanted medical devices or wound dressings, silver has been shown to be absorbed into the systemic circulation, mostly in conjugation with protein and then deposited in human tissues, with higher levels in skin, kidneys, eyes, brain, liver, and bone marrow [51]. Argyria is a rare cutaneous condition resulted by excessive or chronic use of preparations containing silver, with the most characteristic symptom being the discolouration of skin into blue or blue-grey, especially in sunlight-exposed areas [52].

### 2.7. Other Antiseptic Agents

(i) Quaternary ammonium compounds (QACs) are cationic surfactants which have both hydrophobic and hydrophilic groups [26]. QACs target cell walls and membranes. They are quickly absorbed, interacting with membrane lipids, thus disrupting cell structure and function or cause denaturation of essential cell proteins, and leaking of cytoplasmic material [26]. The antimicrobial activities of QACs are governed by their chemical structure and the type of formulation with activity being impacted by fatty substances or anionic surfactants. For example, benzethonium chloride (BZT) is used as a topical anti-infective and an antiseptic effective against bacteria, fungi, moulds, and viruses [53]. Further, benzalkonium chloride (BZK) is used widely as antimicrobial preservative or biocide surfactant, and it is especially commonly found in ophthalmic solutions [54]. BZK displays broad-spectrum activities against bacteria, fungi, virus, algal, but not endospores [55]. The widespread use of BZK has been reported to contribute to the increase in antibiotic resistance concerns [56]. Cetylpyridinium chloride (CPC) demonstrates antiseptic behaviour against Gram-positive pathogens and yeasts but has no effect on Gram-negative microorganisms and mycobacteria. CPC is commonly found as an active ingredient in mouthwashes, toothpastes, lozenges, or mouth sprays for treating minor mouth and throat infections [57,58].
(ii) Octenidine dihydrochloride (OCT) is also a cationic surfactant, belongs to the bipyridine group [59] and has been reported for a wide range of applications such as preoperative skin preparations, prevention, and treatment skin and wound infections [60]. Its spectrum of activity covers both Gram-positive and Gram-negative pathogens including MRSA [60]. Octenidine reduced high-level mupirocin-resistant *Staphylococcus aureus* isolates in vitro by more than 7log cycles at concentrations as low as 0.001% *w/w* within only 30 s [61]. Similar findings were reported for multidrug-resistant Gram-negative bacteria [62]. Octenidine dihydrochloride (0.1%) with 30% *v*/*v* 1-propanol and 45% *v*/*v* 2-propanol was more effective than 74% *v*/*v* ethanol with 10% *v*/*v* 2-propanol for eradication of skin colonization in central venous catheter sites over 24 h [63]. Moreover, octenidine was highly effective in reduction of infections associated with biofilm formation on orthopaedic implants infections, compared to gentamicin [64].
(iii) Polihexanide (PHMB) is a biguanide antiseptic whose chemical structure is similar to chlorhexidine [65]. The positively charged molecular species interacts electrostatically with the negative-charged lipopolysaccharide compounds of bacterial cell membrane, leading to the leakage of intracellular components; therefore, PHMB can be effective on both Gram-negative and Gram-positive pathogens [66]. Octenidol^®^ and ProntOral^®^ mouthwashes, which contain octenidine and polyhexamethylene respectively, displayed similar antimicrobial potency as 0.2% chlorhexidine digluconate in eliminating *Streptococcus sanguinis, Streptococcus mutans*, *Candida albicans,* and *Fusobacterium nucleatum* [67]. Additionally, both 0.02% PHMB and 0.05% OCT were superior than NaCl 0.09% *w*/*v* solution in removal of biofilms of *Pseudomonas aeruginosa* [68]. PHMB is well tolerated when applied to both skin, and wounds [69].

## 3. Topical Antiseptic Formulations

Scopus, Google Scholar, Science Direct, and PubMed databases were used to search the literature with keywords; “Topical formulation” OR “Transdermal formulations” OR “Topical antiseptic formulations”. The results were further filtered with only original research articles written in English language selected. These were then divided into traditional and advanced antiseptic formulations groups, and their details have been discussed in the following sections.

### 3.1. Traditional Antiseptic Formulations

In this section traditional antiseptic formulations intended for topical delivery will be discussed and a summary of these studies is presented in Table 1.

#### 3.1.1. Solutions

**Figure 2:**
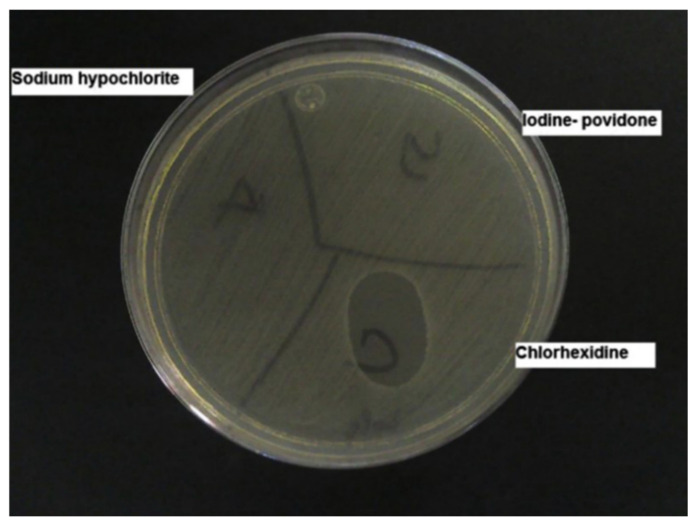
Agar plate in which the substantive effect can be seen. The plate was divided into 3 zones; in each one an antiseptic was tested. Only the zone in contact with skin washed with chlorhexidine showed an inhibition zone. Reproduced with permission from [71], American Jornal of Infection Control, 2013.

Comparisons of antiseptic performance of solution formulations are relatively well reported [91]. Particularly, in a two-step study, 2% *w*/*v* chlorhexidine gluconate in 70% *v*/*v* isopropyl alcohol was proven to have more substantive efficacy against organisms from the skin of human volunteers compared to 10% *w*/*v* sodium hypochlorite and 10% *w*/*v* povidone-iodine [71] (Figure 2). Similarly, it demonstrated a longer-lasting residual effect than triclosan (1% *w*/*v*) in 70% *v*/*v* IPA, making it more suitable than other antiseptics for procedures such as catheter insertion or surgery [33].

**Figure 3:**
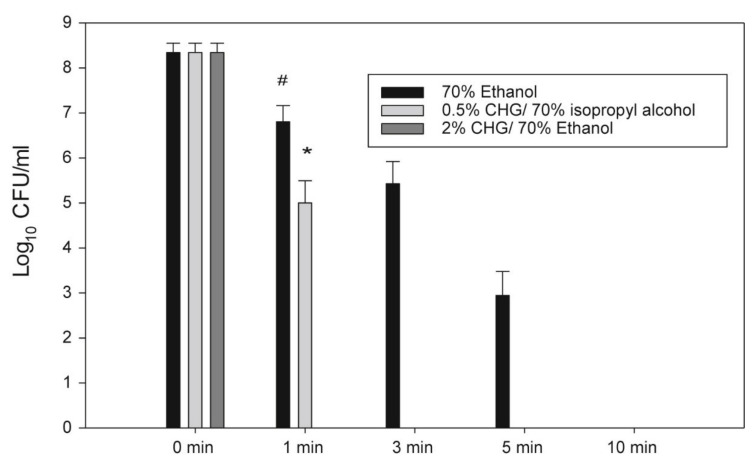
Antiseptic efficacies ethanol and CHG in ethanol solutions. The 2% CHG in 70% ethanol eliminated the MDRAB-Bs completely at the 1 min time point. The 0.5% CHG in 70% isopropyl alcohol eliminated the MDRAB-Bs completely at 3 min time point. However, the70% ethanol eliminated the MDRAB-B completely at 10 min time point. * Indicates significantly lower MDRAB CFUs treated with 2% CHG in 70% ethanol agent than 0.5 CHG in 70% isopropyl alcohol. (Three-way Analysis of Variance (ANOVA) with Scheffe’s post hoc test, *p* < 0.005). #. Indicates significant lower multidrug-resistant *Acinetobacter baumannii* colony forming units (MDRAB CFUs) treated with 2% CHG in 70% ethanol agent than 70% ethanol agent. (Three-way ANOVA with Scheffe’s post hoc test, *p* < 0.005). Reproduced with permission from [72], Journal of Microbiology, Imunology and Infection, 2018.

Chlorhexidine gluconate 2% *w*/*v* in 70% *v*/*v* ethanol was effective in eradicating multidrug-resistant *Acinetobacter baumannii* with biofilms (MDRAB-Bs) with no MDRAB-Bs detected after only 1 min of contact (Figure 3) [72].

On the other hand, according to Koburger et al. (2010), with reference to minimum inhibitory concentration (MICs) and minimum bactericidal concentration (MBCs) values, the antimicrobial effect of polyhexanide and octenidine were deemed to be greater than chlorhexidine digluconate, PVP-iodine and triclosan against the tested microorganisms. In a quantitative suspension test (to determine the minimal concentrations to achieve at least a reduction of 3.8 log cycles for *C. albicans* and 4.8 logs for *S. aureus* and *P. aeruginosa*), octenidine was more effective than triclosan at all-time points [92]. Another recent clinical trial found that 70% isopropyl alcohol solution was equivalent to 2% chlorhexidine gluconate in 70% IPA for skin antisepsis [83], supporting the use of cheaper antiseptics like alcohol [83]. Furthermore, it was found that the simultaneous application of 10% *w*/*v* PVP-I and a topical antiseptic, Alkosol^®^ (96% ethanol, 30 g isopropanol, and 0.1 g ortophenilphenol), in a two-step pre-operative procedure, reduced the extent of surgical site infections as only 6% of included patients had at least one symptom of inflammation after 24 h of surgery, compared to 40% for PVP-I alone [84].

Bashir et al. reported that addition of a film-forming polymer such as an acrylate to a pre-operative solution preparation of 2% chlorhexidine in 70% isopropyl alcohol effectively reduced bacterial colonization in an ex vivo model. This was due to the sustained presence of CHG on the skin surface, thus potentially leading to more sustained antimicrobial activity in prevention of surgical site infections [70].

A topical povidone-iodine solution was employed in a phase 2 trial for the treatment of cancer therapy-related paronychia—an acute nail infection caused by targeted and cytotoxic remedies. Twice daily application of 2% PVP-I solution had a positive effect on clinical outcomes and quality of life [80].

#### 3.1.2. Patch Formulations

A novel mucoadhesive buccal patch which comprised matrix-forming polymers low methoxy amidated pectin (AMP) and 20% *w/w* Carbopol (CAR) was loaded with 4 mg of triclosan. The patch also included β-cyclodextrin-epichlorohydrin polymer (EPIβCD) and anionic carboxymethylated β-cyclodextrin-epichlorohydrin polymer (CMEPIβCD) to improve triclosan (TCS) solubility, as well as its release from the patch. The TCS-EPIβCD complex did improve solubility, compared to a TCS-parent β-cyclodextrin complex although the presence of 1% (*w*/*v*) AMP compromised the complexation and solubilizing properties of both polymeric β-cyclodextrin derivatives (CMEPIβCD and EPIβCD). In addition, the buccal patches formulated with TCS- EPIβCD in combination with AMP-CAR 80:20 (*w/w*) provided immediate and stable drug release and efficacy against *Streptococcus mutans* isolated from the oral cavity [74].

In 2015, a similar study assessed the capability of the polysaccharide psyllium to control the release rate of chlorhexidine from a buccal muco-adhesive patch for local periodontal application. Combining semi-synthetic polymers including sodium carboxymethyl cellulose and hydroxypropyl methyl cellulose (HPMC) with psyllium had the advantages of providing zero-order kinetics for drug release and effective antimicrobial activity against Gram-positive and Gram-negative bacteria [73].

Eudragit^®^ RL 100 was used as the gel-forming agent in chlorhexidine-based medicated dermal patches. Eudragit^®^ RL 100 is a complex made up of “ethyl acrylate, methyl methacrylate and low content of methacrylic acid ester with quaternary ammonium groups” [93]. The amount of quaternary ammonium groups in the RL type is greater than other Eudragit polymers, rendering it more permeable [94,95]. It is widely used as a drug vehicle, controlled release agent, film former, bioadhesive material or suspending agent [96]. Typically, the dermal patches containing Eudragit^®^ RL 100 exhibited efficacious activity against the tested microorganisms [36].

#### 3.1.3. Gels

Gels, along with creams and ointments, are common semisolid formulations used for dermal applications [97]. Gels may be spread easily and offer a cooling effect as a result of solvent volatilization after application [98]. They can be categorized into hydrogels and organogels; hydrogels mainly include water in the liquid phases, while organogels comprise organic solvents [99]. Furthermore, the term “emugels” (as emulsified gels) is used to refer to biphasic systems which encompass a dispersed aqueous gel and a lipid base. Emugels were developed in order to enhance the occlusive characteristics of gels [97].

A thiolated povidine–iodine complex was developed with the intention of enhancing mucoadhesive properties. The gel-forming ability of thiolated PVP and thiolated PVP-I on contacting the mucosal surface and the mucoadhesive features were assessed. Both the thiolated PVP and thiolated PVP-I complex demonstrated merits, such as increasing viscosity and improving the mucoadhesion, as well as controlling iodine release from the gels, compared to unprocessed PVP and PVP-I complex [81].

#### 3.1.4. Lotions

Lotions are utilized particularly (but not popularly in clinical applications) as topical formulations of active substances (i.e., antibiotics, antiseptics, or corticosteroids), intended for treatment of localized cutaneous disorders [98,99]. Moreoever, lotions are more easily applied to sizeable skin areas than more viscous creams or ointments [99].

An aqueous antiseptic lotion containing benzethonium chloride (BZT) at 0.2% was reported to have a rapid and wide-spectrum antimicrobial efficacy equivalent to 76% *v*/*v* ethanol [86] when was tested according to standard Time-Kill protocols [100]. Combined with its known persistence and low propensity for skin irritation, a BZT-aqueous based antiseptic product has advantages over alcohol-based formulations [86,101].

#### 3.1.5. Ointments

Ointments are often selected for their tenacity on the skin to extend a drug’s therapeutic activity over a long time as well as producing a protective layer covering the sites of application. However, they can be associated with irritation due to their occlusive nature arising from their tallowy characteristics [98].

The combination of ointment and body wash containing tea tree oil at 4% and 5%, respectively, was reported to be better than a conventional regime consisting of 2% mupirocin nasal ointment and triclosan body wash for prevention of MRSA-induced infections [90].

An in vitro study tested the PVP-I ointment at numerous concentrations (both standard and diluted concentrations) versus six others antiseptic preparations and a silver-based wound dressing, in terms of eliminating biofilms of *Pseudomonas aeruginosa, Candida albicans*, and MRSA. Following treatment with PVP-I ointment at all concentrations, there were no viable biofilms of *P. aeruginosa* detected after 4 and 24 h. Additionally, PVP-I ointment containing 10% *w*/*v* active PVP-I was deemed effective at eradiating biofilm materials *of C. albicans* and MRSA at both 4 and 24 h following application and performed better than the other tested antimicrobial agents [77].

#### 3.1.6. Creams

There are two main types of cream, oil in water and water in oil creams, of which, o/w cream is more popularly utilized to produce a local effect in case of external disorders, for instance, skin and wound infections [98].

A therapeutic regime of tea tree oil comprising tea tree oil 10% cream and tea tree oil 5% body wash was proposed for eradicating MRSA colonization. There was no significant difference with the standard therapy of 2% mupirocin nasal ointment, 4% chlorhexidine gluconate soap, and 1% silver sulfadiazine cream [88].

#### 3.1.7. Washes/Rubs

The FDA defined antiseptic washes, also known as antibacterial soaps, as products used with water and are rinsed off after use, including hand washes, hand soaps and body washes [102]. Antiseptic rubs (also called hand “sanitizers,” or antiseptic wipes) are substituted when soap and water are inconvenient; they are left on and there is no need to rinse with water [102].

Four different hand wash and hand rub formulations of PVP-I, including 4% PVP-I skin cleanser, 10% PVP-I solution, 3.2% PVP-I in 78% alcohol, and 7.5% PVP-I surgical scrub were compared in a suspension test against Ebola virus (EBOV) and modified vaccinia virus Ankara (MVA) in vitro. Viral titres of MVA and EBOV were reduced by more than 99.99% under both clean environments (0.3 g/L bovine serum albumin; BSA) and contaminated environments (3.0 g/L BSA with 3.0 mL/L erythrocytes) within 15 s of exposure. Among those products, PVP-I solution in an alcohol mixture of 2-propanol and ethanol was the most efficacious at early timepoints. PVP-I could have an important role in limiting diseases related with Ebola, especially in combination with alcohol [78].

Glycerol, which is often used as a humectant, can restrict the clinical effect of pre-operative hand rubs of isopropanol. A hand rub preparation based on isopropanol without glycerol, comprising a combination of ethylhexylglycerin, dexpanthenol, and a fatty alcohol, was more effective in eradiating skin pathogens than the product containing glycerol [103].

Triclosan is one of the most popular antimicrobial agents used in soaps. However, a systematic literature review indicated that triclosan based soaps, used at the concentrations commonly found commercially (0.1–0.45% *w*/*v*), were not more efficacious in preventing infections than non-antimicrobial soaps [89]. The effectiveness of triclosan in antibacterial soaps was tested against twenty isolated strains proposed by FDA [104] either in vitro or in vivo. It was found that antibacterial soaps containing 0.3% *w/w* triclosan did not show a superior effect compared to plain soaps under experimental conditions. This could be a consequence of a short exposure time, or the impact of surfactants in soaps like sodium laureth sulphate on diminishing the bactericidal activity of triclosan [75]. This result led to an US FDA ruling issued in 2013 that all consumer antiseptic wash products need to have demonstrable clinical benefit prior to commercialization, in comparison to plain soap and water [104]. Moreover, the latest FDA ruling released at the end of 2019 announced that three active antiseptic ingredients, benzalkonium chloride, alcohol (ethanol or ethyl alcohol), and isopropyl alcohol are not suitable for use as consumer antiseptic rubs [105].

In contrast, the antifungal and antibacterial effects of a medical triclosan-based shampoo was tested against five isolated microorganisms. Based on the inhibition zones, at all concentrations diluted from original concentration of 0.3% *w/w* (from 10% to 90%), the shampoo had efficacious antimicrobial activity against all three fungal species and one bacterial species (*E. coli*), but no effect on *Staphylococcus aureus*. Generally, antimicrobial shampoos, (e.g., triclosan), have shown efficacy in preventing and treating skin and scalp disorders, such as dandruff whose major cause is *Malassezia globose* [76]. An antiseptic soap with tea tree oil at 0.3% exhibited a similar efficacy in eliminating *E. coli* load on hands as a soap containing triclosan at 0.5% [87].

### 3.2. Advanced Pharmaceutical Formulations

Nanocarriers are colloidal drug delivery systems comprising dispersed particles with diameters less than 500 nm [106]. Nanocarriers have potential applications for parental, oral, dermal and transdermal administration routes [106]. They have been reported to present some merits over conventional preparations such as ameliorated bio-distribution and pharmacokinetics, enhanced therapeutic potency, minimized toxicity, controlled release, increased bioavailability, or drug delivery to target destinations [107,108].

The following sections review the published studies using nanotechnology for delivery of antiseptic agents (key findings are also summarised in Table 2).

#### 3.2.1. Nanoemulsions

Nanoemulsions are transparent or translucent emulsion systems with droplet sizes below 500 nm [107]. These colloidal systems can carry effectively both hydrophilic and hydrophobic drugs into the skin [107]. Compared to traditional topical preparations like gels, creams and ointments, nanoemulsions have been reported to enhance permeation through the skin [126].

A topical o/w nanoemulsion containing cetylpyridinium chloride demonstrated activity against a range of pathogenic fungi, including *T. mentagrophytes, T. rubrum, E. floccosum, Trichophyton tonsurans,* and *Microsporum* spp. as well as 12 species of hyphaes. Furthermore, it was more active against azole-resistant *C. albicans*, and azole-susceptible yeast, compared to other antifungal agents [122]. A benzalkonium chloride loaded nanoemulsion formulation prepared using a high shear homogenization method demonstrated efficacious activity against methicillin-resistant *Staphylococcus aureus* in vitro in mouse and porcine infected wound models. It promoted wound healing as a consequence of reducing inflammation within deep dermal layers and proinflammatory cytokine levels [121]. The formulation had previously been shown to reduce both bacterial colonisation and symptoms of inflammation in burn wounds [127].

Triclosan based nanoemulsions (NEs) were prepared by high shear homogenization followed by probe ultrasonication and using a range of different concentrations of olive oil (OO) and eucalyptus oil (EO) to dissolve TCS. TCS-loaded NEs containing EO had benefits over OO and solutions, in terms of both physicochemical properties and skin permeation ability. Similar results were found with nanoemulsions of CHG, as the inclusion of EO increased penetration into the skin, consequently improving drug retention for localised action. Thus, there are opportunities for nanoemulsions for both dermal hydrophilic and hydrophobic drug delivery [112]. A nanoemulsion of tea tree oil (TTO), prepared using a highspeed homogenizer, produced wider zones of growth inhibition against all isolated microbes than that available gel products with no observed skin irritation [119].

**Figure 4:**
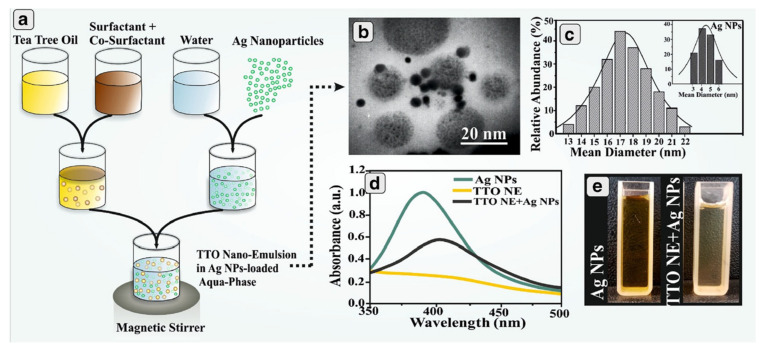
Schematic illustration of preparation procedure of TTO NE + Ag NPs (**a**), Transmission electron micrograph and size distribution of TTO NE and Ag NPs as inset (**b**,**c**). UV–Vis spectroscopy of TTO NE + Ag NPs and Ag NPs, as well as TTO (**d**). Optical images of Ag NPs and TTO NE + Ag NPs (**e**). Reproduced with permission from [120], AAPS PharmSciTech, 2018.

It was reported that there was no serious toxicity caused by a tea tree oil nanoemulsion incorporating silver nanoparticles. TTO NE was prepared by a low energy method using Tween 80 and Span 80 while Ag NPs were prepared using sodium borohydride as a reducing agent and sodium citrate as a stabilizer. This combination demonstrated antibacterial activity against selected microorganisms (from 90 to 95%) at the highest concentration tested (14 μg/mL). Further, blending Ag NPs into a nanoemulsion (the operating process is shown in Figure 4) led to synergistic activity against clindamycin-resistant *E. coli* and an additive influence on *S. aureus* [120]. Thyme oil nanoemulsion, prepared by an ultrasonication method, was loaded into chitosan–alginate polyelectrolyte complex (PEC) via a casting/solvent evaporation method. These PEC films could limit the growth of both Gram-negative *E.coli* and Gram-positive *S. aureus* bacteria 135].

#### 3.2.2. Nanogels

Nanogels are nanoscale three-dimensional hydrogel globules made up of physically or chemically cross-linked hydrophilic polymer networks [128]. When nanogels are applied as dermatological preparations, the hypothesis is that the entrapment of nanoparticles in the gel matrix will extend exposure times on the skin and as a result, extend the duration of therapeutic potency [126].

**Figure 5:**
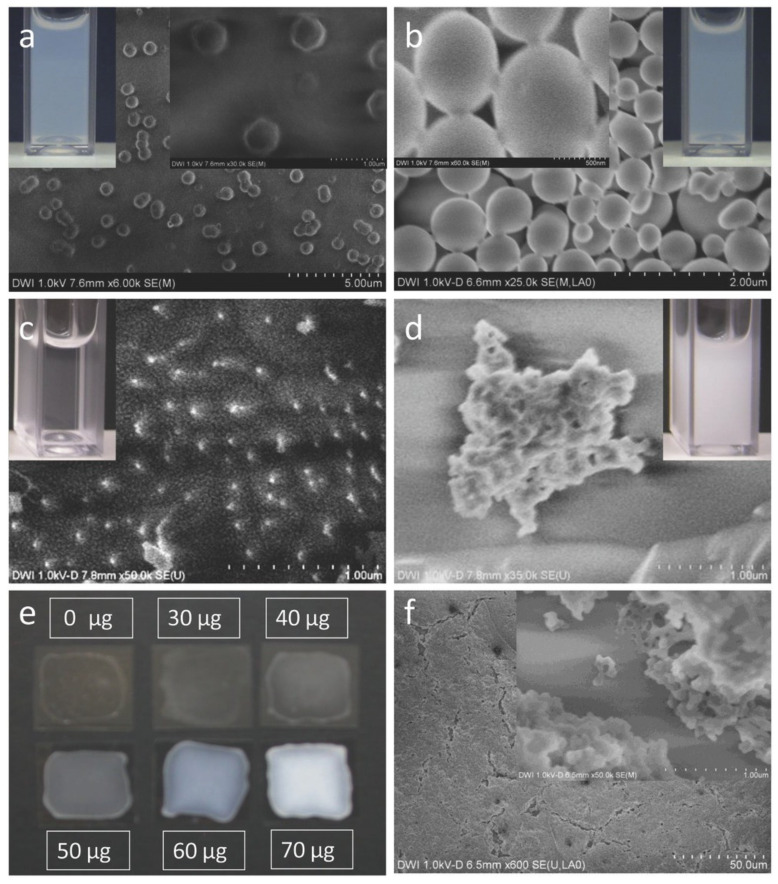
**Field-emission-scanning electron microscope** (FESEM) images of 0.24 mmol β-CD-MA (DS2) nanogels before (**a**) and after complexation with 70 μg mL^−1^ chlorhexidine (CHX) on aluminum surface (**b**). Cryo-FESEM image of 0.47 mmol β -CD-MA (DS4) nanogels before (**c**) and after complexation with 70 μg mL^−1^ CHX (**d**). The inset in (**a**–**d**) shows a dispersion of the β-CD-MA nanogels in a cuvette. Photography of 0.47 mmol β-CD-MA (DS4) nanogels with different CHX content coated on glass plates (**e**) and FESEM images of the nanogel film consisting of the 0.47 mmol CD-MA (DS4) nanogels with 70 μg mL^−1^ CHX (**f**). The second insets in (**a**,**b**,**f**) show enlarged images of the nanogels. Reproduced with permission from [111], Macromolecular Bioscience, 2017.

Chlorhexidine was incorporated into poly(methyl methacrylate) (PMMA) nanogels with α-, β-, or γ-cyclodextrin methacrylate (CD-MA). Field-emission-scanning electron microscope (FESEM) images are shown in Figure 5. This technique enabled chlorhexidine base (CHX) to be entrapped within the nanogel network and, owing to the presence of CD-MA, CHX was released slowly from the material surface into aqueous solution and PBS buffer systems due to decomplexation and redispersion of particles. The inhibitory activity of chlorhexidine base on the growth of *S. aureus* emanated from not only the nanogel surface, but also the aqueous environment [111].

Magnetic nanogels containing cobalt iron oxide nanoparticles were developed for the purpose of controlling pH-related release of CHG. It was found that that the magnetic nanogel was pH-responsive and its electroactivity increased at alkaline pH values. In addition, chlorhexidine was most active and was optimally released at pHs from 6 to 7, i.e., when it is ionized. Therefore, it was proposed that these nanogels would be useful for burns treatment as the pH of the environment is higher than normal [109].

#### 3.2.3. Nanoparticles

The inhibitory effects on autotrophic and heterotrophic microbial growth by silver nanoparticles (Ag NPs), silver ions and silver chloride colloids were assessed by Choi et al. (2008). According to the results of a short-term existent respirometry appraisal, at 1 mg/L silver, silver nanoparticles had a much greater influence on prohibiting nitrifying microbe growth than other forms. Based on an automatic microtiter appraisal, at silver content of 4.2 µM, Ag ions inhibited completely the growth of *E. coli*. None of three silver forms caused cell membrane lysis at 1 mg/L Ag [85]. Colloidal silver formulations encompassing silver nanoparticles were effective against both Gram-positive and Gram-negative pathogens and excellent fungistatic properties were also reported after 7–14 days contact with the silver colloids, especially in case of systems using poly (N-vinylpyrrolidone) and Na-lauryl sulfate as stabilizers [129].

The antiseptic efficacy of an oil-in-water emulsion containing nanoparticles of poly-hexamethylene biguanide hydrochloride (PHMB) was found to be more immediate and long-lasting on human skin colonies in comparison with PHMB solutions, with the duration of effect extending up to 150 min [123].

**Figure 6:**
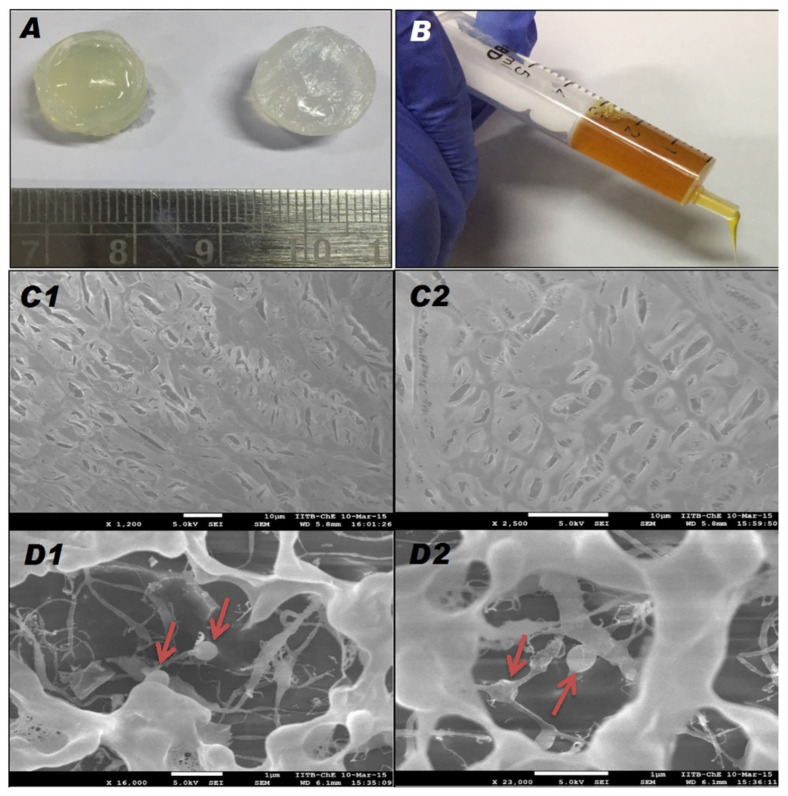
(**A**) Photograph of CNPs-PI-Alg (left) and Blank-Alg gel (right), (**B**) Injectability of CNPs-PI-Alg gel, FEG-SEM images of (**C1,C2**) Blank-Alg gel and (**D1**,**D2**) CNPs-PI-Alg gel; red arrow indicates CNPs. Reproduced with permission from [79], International Journal of Biological Macromolecules, 2018.

A topical alginate gel (Alg gel) (Figure 6) containing PVP-I and vancomycin-loaded chitosan nanoparticles (CNPs) was developed in order to impede and treat orthopedic implant associated infections (OIAIs) [79]. This formulation displayed sustained release of active compounds at the specific sites as well as good biocompatibility and hemocompatibility. Furthermore, this study indicated beneficial antibiofilm and antibacterial activity against *Staphylococcus aureus*, which is the key cause of OIAIs [79].

Nanoparticles containing TCS for the treatment of acne were found to penetrate rapidly into hair follicles and provided a controlled and targeted transport of the antiseptic. Permeation studies found that nanoparticles and emulsions had similar permeation ability albeit lower than a control solution, but retention of TCS in the skin was similar for solution and nanoparticles and highest for emulsion formulations [116].

Solid lipid nanoparticles of triclosan were prepared for topical skin application using glyceryl behenate (GB) and glyceryl palmitostearate (GP) lipids [112]. Solid lipid nanoparticles provide a hydrophobic lipid network for drugs with low aqueous solubility [108]. Overall, solid lipid nanoparticles prepared with GP presented more advantages than with GB, such as smaller size, higher TCS loading, better permeation ability through skin (at 5% concentration of GP), and more TCS retained within the skin [112].

Another formulation approach to ameliorate issues with the relative hydrophobicity of triclosan was to incorporate branched deblock copolymers as stabilizers in the nanoencapsulation process. Three different amphiphilic branched di-block copolymers were synthesized via the copolymerization of a vinyl monomer (butyl methacrylate, styrene, or N-isopropylacrylamide) and a covalently cross-linked core. The obtained triclosan nanoparticles presented a sixfold higher antimicrobial efficacy against *Candida albicans* than triclosan solution [115].

Polymeric nanoparticles (PNPs) are solid, nanostructures colloidal particles with sizes of 10–100 nm produced using biodegradable polymers such as polylactide-polyglycolide copolymers, and polycaprolactones, or natural polymers, such as gelatine, albumin, and collagen [130]. PNPs are generally classified into two types: nanospheres and nanocapsules. Nanocapsules are composed of an outer solid polymeric membrane encapsulating an inner liquid core of oil or water in which the drug is dispersed whereas in nanospheres, actives are enmeshed within the polymer matrix structure [108].

Triclosan was encapsulated into poly L-Lactide (PLLA) nanoparticles (at loadings of 10%, 30%, and 50% *w/w*) by an emulsification–diffusion method and were shown to inhibit bacterial growth thus potential applications in the personal care and surgical implant products, drug delivery systems and wound dressing were proposed [114].

**Figure 7:**
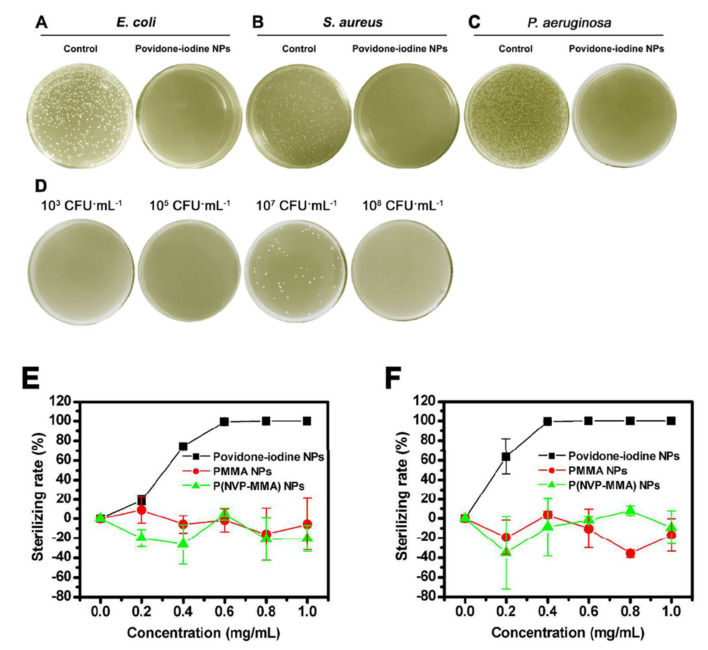
Photographs for the bacterial culture plates of *E. coli* (**A**), *S. aureus* (**B**), and *P. aeruginosa* (**C**) upon a 30 min exposure of povidone-iodine NPs. (**D**) Photographs for the bacterial culture plates of *E. coli* with different concentration upon a 30 min exposure of povidone-iodine NPs. Effect of povidone iodine NPs concentration on their antibacterial activity against *E. coli* (**E**) and *S. aureus* (**F**). Reproduced with permission from [131], ACS Publications, 2017.

Polymeric nanoparticles (NP) containing PVP-I were fabricated using a surfactant-free emulsion copolymerization followed by an iodination procedure. The nanoparticles eliminated 100% of the isolated organisms, including *E. coli* and *S. aureus,* and *P. aeruginosa* (Figure 7) and the decreased hydrophobicity enabled the PVP-I to be amalgamated into conventional products like glue, ink, or dye [131].

#### 3.2.4. Nanocapsules

**Figure 8:**
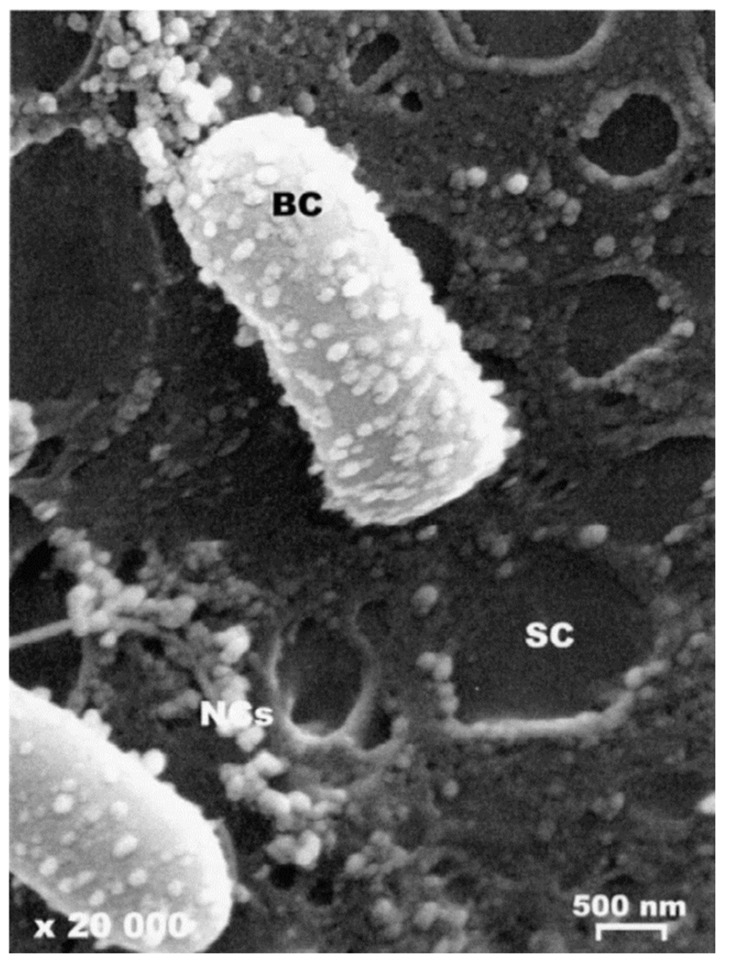
Scanning electron micrographs of 0.6% chlorhexidine base loaded PCL nanocapsules localization on stratum corneum-associated bacteria. Drug loaded nanocapsules adsorbed on bacteria membrane (BC). Reproduced with permission from [110], Journal of Controlled Release, 2002.

Chlorhexidine base was encapsulated into poly(epsilon-caprolactone) (PCL) nanocapsules. In an ex vivo study, after 8 h incubation, the number of colony forming units (CFUs) from skin treated for 3 min with chlorhexidine nanocapsules was notably lower than that of skin treated with CHG solution. Furthermore, residual chlorhexidine from nanocapsules remaining in the stratum corneum was three-times greater, compared to a solution control. The effective adsorption of PCL nanocapsules on the bacterial membrane is shown in Figure 8. Specifically, nanocapsules were found in porcine skin follicles and this resulted in sustained action against *Staphylococcus epidermidis* [110].

Nanoemulsions and nanocapsules containing 10 mg/mL TTO were evaluated in two different infectious nail models. Generally, the nanosystems were effective at reducing the growth of *T. rubrum* which was evidenced through the significant diminution of microorganism count as well as the smallest zones of *T. rubrum* growth after exposure. Particularly, compared to the nanoemulsion, the tea tree oil nanocapsules were more efficacious against fungi [117]. Further studies incorporated these TTO loaded nanosystems into hydrogel preparations. Based on the results of in vivo studies, hydrogels comprised of TTO nanocarriers reduced inflammation caused by UV-B radiation and in the wound healing process, with the most effective being TTO nanocapsule hydrogels [118].

Nanocapsule formulations have been proposed to address increasing antimicrobial resistance. Triclosan nanocapsules were formulated by interfacial deposition and used chitosan as a coating layer and α-bisabolol as an oily core. Positively charged chitosan was included to optimize interaction with negative charged microorganism membranes and α-bisabolol was selected for its ability to disperse lipophilic drugs such as triclosan. Resultant MICs of nanocapsules coated with chitosan were lower than other formulations and the chitosan-coated nanocapsules were incorporated into wound dressings where they were shown to extend the duration and extent of antimicrobial activity [113].

#### 3.2.5. Other Novel Pharmaceutical Formulations

A novel formulation comprising phospholipid (Phospholipon90G) and octenidine dihydrochloride was developed as an alternative for phenoxyethanol, which is often added as solubility enhancer for octenidine but may cause irritation, especially on the mucosae and open wounds). According to an antiseptic efficacy test, the lipid-based formulation had a similar inhibitory potency as a marketed product Octanisept^®^, but had potentially wider application due to the elimination of phenoxyethanol from the formulation [124].

Liquid crystalline systems (LCS) of glyceryl monooleate (GMO) and water were developed as delivery systems for PHMB and cetylpyridinium chloride (CPC). The authors found that the inclusion of the active drugs into LCS affected the drug release, but not the creation of the liquid crystalline phases. Because of the interaction between CPC and GMO, the drug was trapped in the matrix and not likely to release into the medium, leading to a deleterious impact on bactericidal activity. In contrast, PHMB was released at a constant rate, thus having prolonged antibacterial activity against tested pathogens. In general, the evidence from this study suggests that the liquid crystalline systems can used as a carrier for PHMB [103].

Advanced drug delivery systems have been increasingly investigated for topical administration, primarily applying numerous forms of nano-technology. These formulations demonstrated superior therapeutic activities in prevention and treatment of skin and wound infections, compared to conventional dosage forms. However, they show promising potential in vitro but there is a lack of data on products moving into clinical trials and onto the market.

The safety profile and potential toxic effects of nanomaterials is not fully understood and risk/benefit ratio has to be considered [107]. Following topical application, the particles need to remain at the site of action and not enhance uptake into the systemic circulation. Skin permeation studies of formulations must confirm that there is limited absorption through the skin, and this may be further complicated by any infection that compromises the natural barrier function of the skin.

Potential toxicity of nanocarriers can also be caused by chemical mechanisms due to the production of reactive oxygen species, dissolution and release of toxic ions, disturbance of electron/ion cell membrane transport activity, oxidative damage through catalysis, lipid peroxidation, and surfactant properties. Meanwhile, the nanoparticle size and surface properties of nanoformulations are considered as physical factors result in toxicological effects. They relate to membrane damage and disruption of membrane activity, and can affect transport processes, protein conformation/folding, and protein aggregation/fibrillation [132]. Specifically, several studies revealed that silver nanoparticles may cause genotoxic and cytotoxic on human cells [133,134,135]. However, the benefits have been demonstrated in vitro and with the growing issues of antimicrobial resistance, there is increasing pressure to use what we already have more effectively.

**Figure 9:**
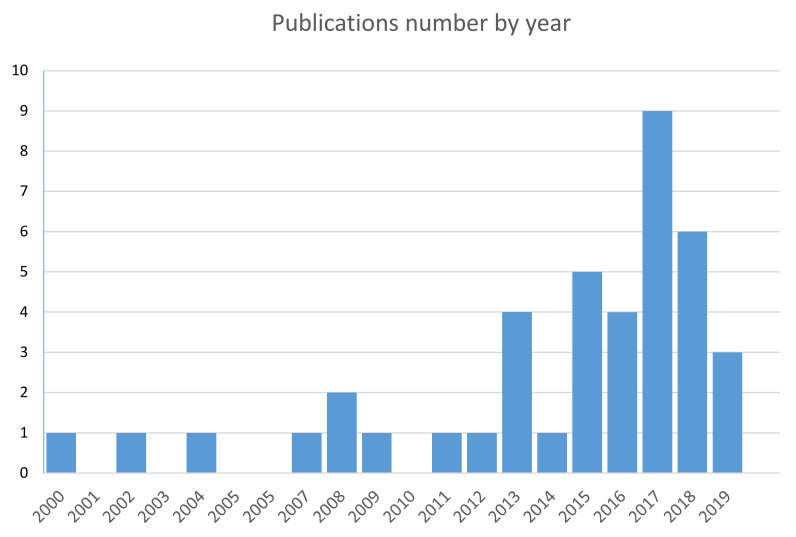
The number of publications on antiseptic formulations for skin and soft tissue infections each year.

Finally, Figure 9 summarises the annual distribution of publications focused on antiseptic formulations included in this review article. As is evident in Figure 9, the there is a large increase in the number of publications after 2013.

## 4. Conclusions

The current review has successfully gathered comprehensive information on various antiseptic formulations employed to prevent and treat skin and soft tissue infections. It is evident from the current review that research in recent years has established topical, mostly dermal, delivery as a promising route. Its ability to bypass the hepatic first-pass metabolism and easy accessibility yet relatively impermeability holds great promise, especially in the treatment of skin infections. This distinctive advantage allows the application of a wide range of external dosage forms that can be easily removed if necessary. These formulations have evolved from simple ointments, creams, and solutions to advanced nanotechnological assisted formulations. However, it is of equal importance that these sophisticated formulations should address clinical and market needs. It is expected that this review will be a helpful resource for formulation scientists to understand and further to develop the antiseptic skin formulations to achieve specific therapeutic objectives.

## Figures and Tables

**Table 1:** Summarised characteristics of traditional skin antiseptic formulations.

Drug	Concentration	Formulation Type	Combination	Carrier Polymer	Manufacturing Technique	Study Characteristics	Reference
Chlorhexidine gluconate (CHG)		Dermal polymeric patch		Eudragit RL100		To characterize properties of developed patches regarding their drug release and antimicrobial activity	[36]
Chlorhexidine gluconate	2% CHG in 70% isopropyl alcohol (IPA)	Solution	Acrylate copolymer			To test the effectiveness of adding a film-forming acrylate copolymer to a topical CHG-based preparation on minimizing CHG loss, compared to a marketed CHG solution	[70]
Chlorhexidine gluconate	2% CHG in 70% IPA	Solution				To contrast the residual effects of 2% CHG in 70% IPA *v*/*v* and 1% triclosan in 70% IPA *v*/*v* on skin bacterial communities	[33]
Chlorhexidine gluconate	2% CHG in 70% IPA	Solution				To compare the antiseptic activity of 10% sodium hypochlorite and 2% CHG in 70% IPA	[71]
Chlorhexidine gluconate	2% CHG in 70% ethanol	Solution				To appraise the desiccation and ethanol resistance of multidrug-resistant *Acinetobacter baumannii* with biofilms (MDRAB-Bs). To compare the antiseptic activities of a combination of CHG and 70% ethanol with 70% ethanol disinfectants used for MDRAB-Bs	[72]
Chlorhexidine base		Mucoadhesive polymer patches		Psyllium and three types of semi-synthetic hydroxypropyl methyl celluose	A casting-solvent evaporation technique	To test the effectiveness of polysaccharide psyllium in the mucoadhesive patches for controlling release	[73]
Triclosan		Methoxy amidated pectin- based mucoadhesive buccal patch		β-cyclodextrin		To develop buccal patches and determine drug release, antimicrobial and in vitro absorption from patches	[74]
Triclosan	0.3%	Soap				To study the in vitro and in vivo antibacterial activity in soap	[75]
Triclosan	0.3%	Shampoo				To assess the antimicrobial efficacy of the shampoo against bacteria and fungi	[76]
Povidone-iodine (PVP-I)	10%	Ointment				To compare the in vitro antibiofilm effect of diluted PVP-I ointment with other six tested products against *P. aeruginosa* and multi-species biofilms of *C. albicans* and MRSA	[77]
Povidone-iodine	4% PVP-I skin cleanser, 7.5% PVP-I surgical scrub, 10% PVP-I solution and 3.2% PVP-I/alcohol solution	Hand wash and hand rub				To study the in vitro potency of four hand hygiene formulations of povidone iodine against Ebola virus	[78]
Povidone-Iodine		Alginate hydrogels	Vancomycin	Vancomycin loaded chitosan nanoparticles (CNPs) by ionic gelation method	Modified ionic gelation method	To assess in vitro release of vancomycin and PVP-I from the hydrogel. To assess the bactericidal and antibiofilm efficacy of hydrogels	[79]
Povidone-Iodine	1% and 2%	Solution				To analyse the effectiveness and safety of 1% or 2% PVP-I topical solution in patients with cancer therapy-associated paronychia during 6–8 weeks.	[80]
Thiolated PVP and Thiolated PVP-iodine complex		Solution	2-(2-acryloyl–Ethyl disulfanyl)-nicotinic acid (ACENA)			To test in vitro mucoadhesive properties and the release of iodine from thiolated PVP-Iodine complexes	[81]
Isopropanol	75% (*w/w*)	Hand rub	Glycerol 0.725% (*w/w*)			To investigate the role of glycerol in pre-surgical hand rub products, based on EN 12791, especially after 3 h of application	[82]
Isopropyl alcohol	70% (*v*/*v*)	Solution				To study the potency of isopropyl alcohol and chlorhexidine in the prevention of blood cultures impurities	[83]
Ethanol	96%	Solution	Isopropanol-30 g and ortophenilphenol-0.1 g			To determine the effect of the combination of 96% ethanol, 30 g isopropanol, 0.1 g ortophenilphenol and PVP-I in minimizing surgical-site infections, compared to that of single use PVP-I	[84]
Silver Chloride		Colloidal solution				To study the suspension potency on the microbial autotrophic and heterotrophic growth	[85]
Benzethonium chloride (BZT)	0.2%	Lotion				To test the antimicrobial efficacy of an ethanol- based antiseptic and water-based antiseptic products containing 0.2% BZT	[86]
Tea tree oil	3%	Soap				To assess the potency of 0.3% *Melaleuca alternifolia* essential oil versus 0.5% triclosan hand soap formulations	[87]
Tea tree oil		Tea tree 10% cream, tea tree 5% body wash				To compare the efficacy of the combination of tea tree 10% cream and tea tree 5% body wash with the standard theory in eliminating MRSA	[88]
Triclosan	0.1–0.45% *w*/*v*	Soap				To evaluate the efficacy of soaps with and without triclosan and investigate potential hazards in the emergence of antibiotic resistance	[89]
Tea tree oil		4% tea tree oil nasal ointment and 5% tea tree oil body wash				To compare the ability to eradicate MRSA between the combination of a 4% tea tree oil nasal ointment and 5% tea tree oil body wash with a standard theory of 2% mupirocin nasal ointment and triclosan body wash	[90]

**Table 2:** Summarised characteristics of advanced skin antiseptic formulations.

Drug	Concentration	Formulation Type	Combination	Carrier Polymer	Manufacturing Technique	Study Characteristics	Reference
Chlorhexidine gluconate	0.2%	Nanogel containing magnetic Cobalt iron oxide nanoparticles		Chitosan and gelatin	Solution casting method	To investigate the release and pH-dependent response of chlorhexidine gluconate from a magnetic nanogel	[109]
Chlorhexidine base		Poly(epsilon-caprolactone) nanocapsules		Poly(epsilon-caprolactone)	Solvent displacement method	To evaluate the antibacterial ability of poly(epsilon-caprolactone) nanocapsules containing chlorhexidine base and the absorption of active into the stratum corneum	[110]
Chlorhexidine base		α-, β-, and γ-cyclodextrin methacrylate (CD-MA) containing poly (methyl methacrylate) (PMMA) based nanogels			CD-MA containing nanogels were synthesized by the radical precipitation polymerization technique	To study the capacity of chlorhexidine base in PMMA nanogels. To assess the bactericidal against *Staphylococcus aureus* of CD-MA nanogels	[111]
Chlorhexidine digluconate		Nanoemulsions	Eucalyptus oil (EO) or Olive oil (OO)		HSH followed by probe ultrasonication	To investigate the drug release, skin permeation and retention of CHG from nanoemulsions. To evaluate impact of methacrylate powder dressing in controlling the CHG release	[112]
Triclosan (TCS)		Chitosan-coated nanocapsule		Poly(epsilon-caprolactone) (PCL)	Interfacial deposition of preformed polymers	To characterize properties of nanocapsule comprised of α-bisabolol and TCS. To study the antimicrobial activity against tested pathogens. To testify the compatibility as incorporating nanocapsule into wound dressings	[113]
Triclosan	10%, 30%, and 50%	Poly-L-lactide (PLLA)/triclosan nanoparticles		Poly-L-lactide (PLLA)	Emulsification–diffusion technique	To evaluate the release of triclosan from PLLA nanoparticles and its antimicrobial activities	[114]
Triclosan	0.5% *w/w*	Nanoparticles stabilized by branched diblock copolymers		Branched diblock copolymers: PEG-*b*-PNIPAM (BDP 1); PEG-*b*-PBMA (BDP 2); PEG-*b*-PSty (BDP 3)	Emulsion-freeze-drying technique	To assess fungicidal ability against *C. albicans* of triclosan nanoparticles	[115]
Triclosan		Nanoparticles		Eudragit E 100	Emulsification–diffusion by solvent displacement method	To compare in vitro percutaneous permeation of nanoparticles containing triclosan, with two commercial formulations used for treating acne, including a solution and an o/w emulsion	[116]
Triclosan		Solid lipid nanoparticles (SLNs)		Glyceryl behenate (GB) and Glyceryl palmitostearate (GP)	Hot high shear homogenisation followed by probe ultrasonication	To investigate the impact of SLNs in delivery of TCS to deeper skin layers and hair follicles and compare the permeation ability of GB-SLNs and GP-SLNs	[112]
Triclosan		Nanoemulsions	Eucalyptus oil (EO) or Olive oil (OO)		HSH followed by probe ultrasonication method	To develop and characterise stable nanoemulsion formulations. To evaluate the ability of NEs in improving skin retention of TCN	[112]
Tea tree essential oil (TTO)	10.0 mg mL^−1^	Nanoemulsions (TTO-NE) and polymeric nanocapsules (TTO-NC)		Poly(e-caprolactone)	TTO-NE by spontaneous emulsification and TTO-NC by interfacial deposition of the preformed polymer methods	To investigate the in vitro fungicidal potency against *Trichophyton rubrum* of TTO-NE and TTO-NC systems	[117]
Tea tree essential oil (TTO)		Hydrogels containing Nanoemulsions (TTO-NE) and nanocapsules (TTO-NC)		Poly(e-caprolactone)	Nanoemulsion: spontaneous emulsification Nanocapsules: interfacial deposition of preformed polymer	To evaluate physicochemical properties of hydrogels and their efficacy in wound healing and protecting skin from UV-B rays	[118]
Tea tree oil (TTO)		Emulgel (EG) containing TTO-loaded nanoemulsion (NE)			Nanoemulsion: High energy emulsification	To evaluate the physicochemical properties, the ex vivo penetration, antimicrobial potency and safety of topical emulgel	[119]
Tea tree oil (TTO)		Nanoemulsions (NE)	Silver nanoparticles (Ag-NPs)			To investigate cytotoxicity as well as antimicrobial ability of the prepared nanoemulsions against clindamycin-resistant *Escherichia coli* and *S. aureus.* To appraise the synergistic effect of TTO NE and Ag NPs against tested microorganisms	[120]
Silver		Silver nanoparticle (Ag NPs)		Polyvinyl alcohol (PVA)		To estimate the suspension efficacy on the autotrophic and heterotrophic growth. To investigate silver species properties	[85]
Benzalkonium chloride (BZK)	0.6% BZK for in vitro studies and 0.2% BZK for in vivo studies.	Nanoemulsion		EDTA	High-energy homogenization using high shear conditions	To evaluate the in vitro and in vivo antimicrobial effect against isolated bacterial species	[121]
Cetylpyridinium chloride (CPC)		Oil in water nanoemulsions				To assess the fungicidal potency	[122]
Polyhexanide (PHMB)	0.05%	nanoparticle-emulsion		Lipofundin^®^ MCT 20%		To compare the efficacy of a particle- and non-particle antiseptic formulations	[123]
Poly-hexamethylene biguanide hydrochloride (PHMB) and cetylpyridinium chloride (CPC)	0.2 and 2.0% (*w/w*) of PHMB0.05 and 2.5% (*w/w*) of CPC	Liquid crystalline systems (LCS)		glyceryl monooleate (GMO)		To investigate the release of PHMB from liquid crystalline systems, and its antimicrobial activity as incorporated into these systems	[103]
Octenidine dihydrochloride	0.1%	Phosphatidylcholine formulation	Soybean phosphatidylcholine (Phospholipon 90G)			To assess the antimicrobial potency of octenidine formulations	[124]
Thyme oil	1,2 and 3% *v*/*v*	Nanoemulsion	Chitosan-Alginate		Ultrasonication	To investigate the potential application of alginate–chitosan polyelectrolyte complexes films containing thyme oil nanoemulsion in wound dressings	[125]
